# Effects of a Gender-Balancing Strategy on Resident Panels in a Primary Care Setting

**DOI:** 10.1007/s11606-024-09075-0

**Published:** 2024-10-16

**Authors:** Samantha Mannion, Andrew J. Halvorsen, Carl Andersen, Emily Leasure, Sara Bonnes

**Affiliations:** 1 Division of General Internal Medicine, Mayo Clinic, Rochester, MN USA; 2 Internal Medicine Residency Program, Mayo Clinic, Rochester, MN USA; 3 Division of Community Internal Medicine, Geriatrics and Palliative Care, Mayo Clinic, Rochester, MN USA

**Keywords:** patient preference, primary care, physician patient relationship, residency, medical education, gender

## Abstract

**Background:**

Patients often prefer gender concordance when choosing a primary care practitioner. In a trainee setting, this may lead to unequal training opportunities for male and female resident physicians. Residency leadership may be interested in ways to promote balance in patient empanelment.

**Objective:**

To assess the efficacy of an intervention to equalize imbalance in patient gender on resident primary care panels.

**Design:**

Observational cohort study.

**Participants:**

Categorial internal medicine residents beginning residency in 2020.

**Interventions:**

The panels of internal medicine residents were manually rebalanced at the beginning of training for a new cohort of residents with the goal of having similar numbers of male and female patients on each resident’s panel.

**Main Measures:**

Panel data was observed for 2 years following intervention. Number of male patients, number of female patients, and overall panel size were compared between male and female residents at baseline, 6 months, and 24 months.

**Key Results:**

The analysis included 28 female residents and 20 male residents. After rebalancing, baseline panels had similar numbers of male patients (median of 50 on both male and female residents’ panels; average panel 54.7% male) and female patients (median of 41.5 on female residents’ panels and 41 on male residents’ panels; average panel 45.3% female). At the end of the follow-up period, a significant difference was observed in the median number of male patients (59.5 and 43.5; *p* < 0.001) and female patients (33.5 and 48.5; *p* < 0.001) between male and female residents, but no difference was observed in overall panel size.

**Conclusions:**

A steady drift towards gender concordance was observed over 2 years following a rebalancing intervention. Program leadership overseeing primary care empanelment for resident physicians may consider periodic rebalancing of panels in addition to other interventions to ensure equal training opportunities and best prepare residents for future practice.

## INTRODUCTION

Patients often display a preference for gender concordance when choosing a physician, particularly in primary care and fields likely to involve sensitive examinations, such as gynecology and urology.^1–5^ There are several possible explanations for this observation, including perceptions of varying communication and practice styles between male and female physicians,^6,7^ cultural and religious factors,^5,8^ increased comfort discussing sensitive topics and/or having a sensitive examination,^4,5,8^ and an impression of easier communication with a physician of one’s own gender.^2^ This preference for gender concordance manifests in both the number of office visits with a same-gender physician^9–11^ and in empanelment among primary care clinicians.^1,12–14^

This observation has additional relevance for resident physicians training in adult primary care fields such as internal medicine and family medicine. A key component of training is serving as a primary care physician (PCP) for a panel of patients during residency. Significant disparities in empanelment by gender may lead to unequal training experiences, particularly regarding gender-specific care such as pelvic examinations.^10,15^ As a result of having fewer patients of the opposite gender on one’s panel and fewer appointments scheduled with these patients, both male and female residents may graduate feeling inadequately prepared to evaluate and manage gender-specific issues.

Additionally, a gendered imbalance in empanelment may contribute to unequal workload between male and female resident physicians, as recently demonstrated in our clinic’s population^16^. Female patients tend to interact with the healthcare system more frequently,^17–20^ and average visit length between female patients and female physicians is longer than any other dyad.^6,11^ Limited data suggests that female patients send messages through the electronic health record (EHR) slightly more often than male patients,^16,21,22^ potentially relevant in that greater time spent answering electronic messages has been linked with higher rates of burnout.^23,24^

One way for program leadership to address this imbalance in empanelment is by manually adjusting panels to create greater similarity between residents. The purpose of this study is to examine the effects of such a gender-balancing intervention, which was implemented for a new class of incoming internal medicine residents at a large academic medical center in the Midwestern United States. To our knowledge, the effects of an intervention wherein panels are intentionally rebalanced with the goal of equalizing gender balance across physicians have not been previously described in either a resident or a faculty clinic, and it is unknown whether such an intervention would provide durable change. Based on historical trends in our clinic^14^ and published literature suggesting a preference for gender concordance in primary care^1,2,4^, we hypothesized that panels would most likely drift back towards gender concordance following the intervention. We sought to quantify trends in panel redistribution and observe the timing of any notable shift to determine the effectiveness of the intervention and its utility for future resident classes.

## METHODS

### Participants and Intervention

All categorical internal medicine residents at our institution are assigned a panel of primary care patients at the beginning of residency. Patients from graduating post-graduate year 3 (PGY-3) residents are transitioned to incoming PGY-1 residents (although there may occasionally be special considerations for transition to a PGY-2 for complex patients requiring a warm handoff). Resident panels of all years are also open to patients new to the health system needing a PCP, or patients may request a change in PCP. To minimize transitions in care, patients are not assigned new PCPs other than due to resident transition/graduation, or patient request. At the time of this study, there were no specialized tracks (such as women’s health or primary care) which may have affected panel makeup. Panels were manually rebalanced effective July 2020 for the incoming cohort of post-graduate year 1 (PGY-1) residents with the goal of assigning each resident a similar number of male and female patients. A similar intervention had not previously been conducted within the residency program. Residents were excluded from the analysis if they did not complete the training program or if panel data was unavailable. Resident gender was recorded from self-identified data. Patient gender was recorded as male or female, as registered in the electronic medical record. Although the medical record did allow for recording of genders other than male and female (for example, non-binary or choose not to disclose), none of patients involved in this analysis chose to identify as such. Panel data, including number of male and female patients and overall panel size, was abstracted at 12 subsequent time points over the 24-month period, culminating in July 2022. As a pre-intervention comparison, panel data from the graduating class of 2020 (whose panels were inherited by the incoming cohort of new residents) were abstracted 6 months prior to graduation and panel rebalancing. This intervention was originally designed as quality improvement initiative; subsequent analysis and publication of the data were approved by the Internal Medicine Research in Education Group and deemed exempt by the Mayo Clinic Institutional Review Board (IRB #23-011512).

### Analysis

Panel size and the percentage of female patients on each panel were mapped at all recorded time points, culminating in July 2022. The number of female patients, number of male patients, and overall panel size were compared between male and female residents at baseline, 6 months, and 24 months. Due to the small sample size, a normal distribution of the data was not assumed, and a non-parametric test was chosen for these comparisons. Wilcoxon rank-sum tests were run first on the baseline data to ensure that the intervention achieved its goal of creating balanced panels. Similar analyses were then repeated at 6 months and 24 months to evaluate for changes in gendered empanelment between male and female residents.

## RESULTS

### Pre-intervention Comparison

**Figure 1:**
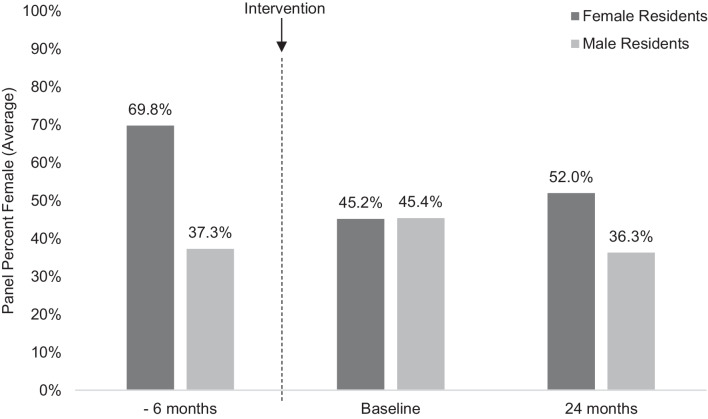
Pre-intervention comparison. The leftmost columns represent panel data from the class of 2020, prior to panel rebalancing in July 2020 when the patients in this cohort were reassigned to the new incoming resident class. The pre-intervention class comprised 39 male residents and 13 female residents; the incoming class comprised 20 male residents and 28 female residents.

In January 2020, prior to rebalancing, female resident panels were, on average, 69.8% female and male residents’ panels were 37.3% female (Figure 1). Between male and female panels, there were significant differences in the median number of female patients (34 and 80, respectively; *p*<0.001) and male patients (60 and 28; *p*<0.001). Median overall panel size in the pre-intervention cohort was also larger for female residents (105 patients) than male residents (96 patients) (*p*<0.001). Of note, the pre-intervention cohort had 39 male residents and only 13 female residents, while the incoming class was somewhat female-predominant (28 female residents and 20 male residents).

### Changes in Empanelment

**Figure 2:**
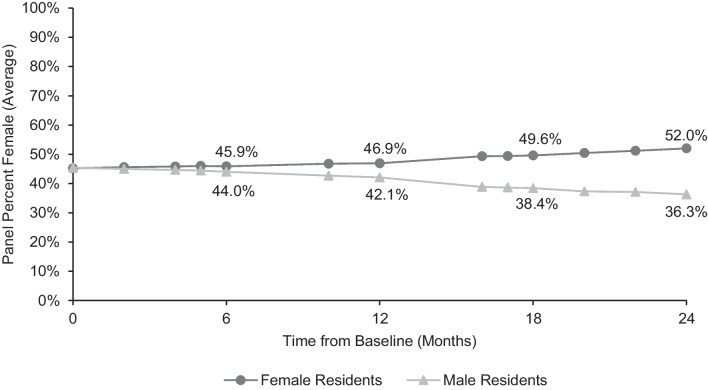
Percentage of empaneled patients who are female. Overall clinic population was 45.3% female at baseline and 45.5% female at 24 months.

There were 53 categorical internal medicine residents in the cohort during the study timeframe. Five were excluded from the analysis due to missing data or a change in enrollment status. The final analysis included 28 female residents and 20 male residents. Following manual rebalancing in July 2020, female residents’ panels were, on average, 45.2% female, and male residents’ panels were 45.4% female. There was no significant difference in the median number of female patients (41 and 41.5; *p* = 0.21), male patients (50 and 50; *p* = 0.86), or total panel size (90 and 91; *p* = 0.21) between male and female residents at baseline (Table 1). Panel data was examined at multiple subsequent time points, demonstrating a steady drift towards gender-concordant empanelment (Figure 2).

**Table 1:** Resident Panels at Baseline, 6 Months, and 24 Months

		Female residents (*n* = 28)	Male residents (*n* = 20)	*p* value
Baseline	Female patients empaneled, median (IQR)	41.5 (1.25)	41 (2)	0.21
	Male patients empaneled, median (IQR)	50 (1.25)	50 (2.25)	0.86
	Total panel size, median (IQR)	91 (2)	90 (1.25)	0.21
*t* = 6 months	Female patients empaneled, median (IQR)	41.5 (2.25)	39 (3)	<0.001
	Male patients empaneled, median (IQR)	49 (2.75)	50 (2.25)	0.03
	Total panel size, median (IQR)	90 (3.25)	89 (3)	0.53
*t* = 24 months	Female patients empaneled, median (IQR)	48.5 (10.5)	33.5 (4.25)	<0.001
	Male patients empaneled, median (IQR)	43.5 (5.25)	59.5 (7)	<0.001
	Total panel size, median (IQR)	93 (4.75)	95 (5.25)	0.18

*p*-values calculated using Wilcoxon rank sum tests

*IQR* interquartile range

At 6 months, female residents had a median of 41.5 female patients compared to 39 for male residents (*p* < 0.001). Female residents had a median of 49 male patients, while male residents had 50 (*p* = 0.03). Overall panel size did not differ significantly between the two groups; female residents had a median of 90 patients and male residents had 89 (*p* = 0.53).

**Figure 3:**
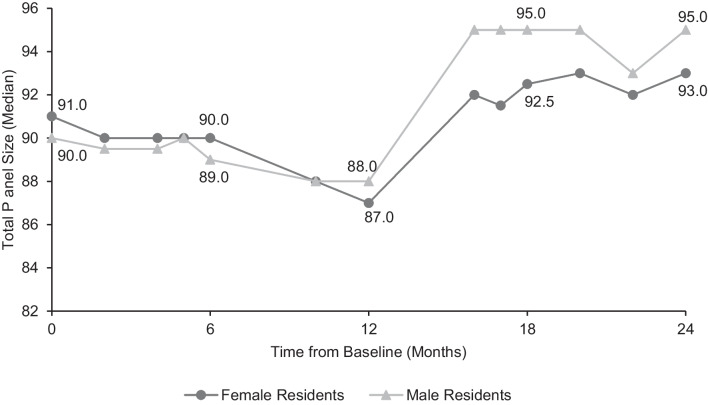
Total panel size. Overall median panel size was 91 patients per resident at baseline and 93 patients per resident at 24 months. Using Wilcoxon rank-sum tests, no significant differences were found in total panel size between male and female residents at baseline (*p* = 0.21), 6 months (*p* = 0.53), or 24 months (*p* = 0.18).

At the end of the 2-year follow up, female residents’ panels were, on average, 52.0% female and male residents’ panels were 36.3% female. Significant differences were observed in the number of female patients (median 48.5 on female residents’ panels and 33.5 on male residents’ panels; *p* < 0.001) and the number of male patients (median 43.5 on female residents’ panels and 59.5 on male residents’ panels; *p* < 0.001). From baseline, female residents had a median of 7 more female patients on their panels at 2 years, and 6.5 fewer male patients. Conversely, the median number of male patients on male residents’ panels increased by 9.5 from baseline, while they had 7.5 fewer female patients. Again, overall panel size did not differ significantly; female residents had a median of 93 patients and male residents had 95 (*p* = 0.18) at the endpoint (Figure 3).

## DISCUSSION

Following an intervention to equalize gender imbalance on internal medicine residents’ primary care panels, a significant drift towards gender concordance was observed over 2 years. Overall panel size did not differ between male and female residents at any of the examined time points. Several hypotheses can be explored to explain the observations, which are likely multifactorial. After receiving notice of PCP change at the beginning of the intervention, there may have been a small number of patient-generated requests upon learning the new physician’s name and presumed gender, although the relatively slow change in empanelment (+/- less than 2 patients of either gender within the first 6 months) suggests that this plays a very limited role, if any. The scheduling process may contribute to gender-concordant care if a patient requests a same-gender physician for a sensitive issue or one is suggested to them based on the scheduler’s conscious or unconscious assumption of the patient’s preferences. While institutional policy does not permit appointment requests based on gender preference or other personal characteristics, there may be variable enforcement of this policy if the appointment is for a “sensitive” issue. Additionally, during the study timeframe, patient online self-scheduling became available for established patients, and this change may have had the unintended consequence of allowing patients to select for more gender concordance in appointments, although it should not have directly impacted empanelment.

Following any type of visit, patients or physicians might request a change in empanelment, particularly if they had a strongly positive (or negative) impression of the care received, if a significant amount of chronic disease management or preventative care was done, and/or if they had not yet met the PCP that was originally assigned to them. While requests based on gender alone are not granted, a patient or physician might request a change based on an overall positive experience, a perception that may be more likely in gender-concordant visits. There is some evidence that gender concordance is associated with more patient-centered care and improved rapport, particularly in female/female dyads;^6,7,25^ studies evaluating the relationship between gender concordance and patient experience have been mixed.^26–29^ Support for this visit-driven change hypothesis is given by the slight increase in the rate of change around July 2021 (12 months after intervention) and a corresponding jump in overall panel sizes for all residents (Figure 3), when many patients began returning to the clinic for in-person visits and routine preventative care after having delayed such care during the early part of the COVID-19 pandemic.

The degree of gender disparity in empanelment at 2 years (52.0% female patients on female panels and 36.3% female patients on male panels) was not as striking as it was prior to the intervention (69.8% female patients on female panels and 37.3% female patients on male panels). Bearing in mind the limitations of this comparison due to changes in the gender makeup of resident classes between the pre-intervention and study cohorts, we can infer that the intervention may have been partially effective in its goal of reducing disparities in empanelment. Ultimately, however, the goal of a residency program is not to shift panel structures but rather to ensure that graduating physicians are skilled in the complete care of patients of all gender identities and backgrounds. The ability to perform a diagnostic evaluation including appropriate history taking and a comprehensive physical examination is part of this training^30^. Examples abound of clinical situations which present differently across genders: due to a higher incidence of “atypical” symptoms (a term which in itself conveys bias), women more often experience delayed diagnosis of acute coronary syndrome^31,32^, and men suffer from missed diagnoses of depression^33,34^. Non-binary and transgender patients may struggle to obtain appropriate contraception, placing them at risk for unintended pregnancy^35^. Other aspects of clinical care, such as preventative services, care during pregnancy and lactation, and prescription drug management, also have gender-specific elements.

Even if panels are perfectly balanced, additional strategies may still be needed to create equal training opportunities, noting that empanelment to a certain physician does not guarantee that this physician will perform gender-specific care. For example, a patient empaneled to a male physician may request a female physician for the appointment when they will have their Pap smear done. An earlier study conducted by Garrison and colleagues found disproportionality in gender-specific visits for both male and female residents despite no differences in empanelment.^10^ Creative solutions, such as rotations in urology or gynecology,^10^ or scheduling of dedicated clinical experiences, may be considered to ensure adequate opportunity to learn these skills.

Should other institutions adopt a similar panel restructuring initiative, we would suggest a timeline that coincides with an existing need for reassignment due to graduation, which is typically on a 3-year schedule for a given patient cohort in internal medicine and family medicine programs. Making use of this existing schedule minimizes disruptions to continuity between a patient and their resident PCP, benefiting both parties. However, given the significant panel shift observed here over only 2 years, spacing out rebalancing efforts any further may be ineffective.

When surveyed, patients often report the gender of the physician as unimportant or less important than other factors such as clinical knowledge, communication skills, and waiting times.^5,36–39^ Given this professed agnosticism towards the gender of the physician, the degree of panel imbalance observed in our data as well as other literature^1,12–14^ may be surprising. While patients may value professional competency more highly than personal characteristics, they often do not have access to this information when scheduling an initial appointment.^37^ Basic demographic information, however, such as age and gender, is readily available to prospective patients, and given the increasing number of female physicians in the workforce, patients can easily select for gender when choosing a PCP while still meeting other logistical criteria that they consider important, such as office location, hours, and types of insurance accepted. Once established with a PCP, many patients may be inclined to continue with that physician. Illustrative of this point is a study by Schnatz and colleagues, who found that 83% of women selected a female gynecologist when no other information was available, but the majority chose a male gynecologist when information to support the humanistic and technical qualities of the male physician was provided.^3^ Earlier survey-based studies may also be limited by patients’ concern for being perceived as biased by expressing a gender preference when completing the survey. This study adds to the existing literature by going beyond theoretical, reported values to illustrate real-world trends through panel data, and our findings support the conclusion that patients do tend to demonstrate a preference for a same-gender PCP.

### Limitations

This was a single-center study conducted with a single cohort of residents, limiting the generalizability of findings. The number of male and female residents varies with each class, which likely would impact findings if a similar study were repeated with other resident classes. In this study, the pre-intervention comparison cohort contained notably more male residents than female residents in contrast with the study cohort which was slightly female-predominant. The relatively low number of female physicians in the pre-intervention cohort may have led to increased density of female patients on their panels and limit the usefulness of this comparison as a measure for the success of the intervention. A limitation of our study population is the absence of patients with gender identities other than male or female, an underrepresentation compared to the general population^40^. Some possible explanations for this include the older age of an internal medicine clinic population, wherein the frequency of individuals who report nonbinary identities is lower^40^, as well as under-reporting of transgender and nonbinary gender identities in healthcare settings due to concerns about privacy and discrimination^41–43^. Finally, the COVID-19 pandemic during the study timeframe had significant effects on healthcare utilization, including greatly expanded use of telehealth services, although did not change empanelment protocols.

## CONCLUSION

Following an intervention to equalize gender imbalance on resident panels, a significant drift towards gender concordant empanelment was observed over 2 years. This has important implications for residency programs seeking to create equal training opportunities, particularly as programs consider expansion of competency-based assessment. Without adequate opportunity to learn certain skills, trainees may struggle to maintain and demonstrate competency. Programs may consider implementing similar periodic rebalancing interventions, as well as exploring other mechanisms to create opportunities for trainees to provide primary care to patients of all backgrounds. Future research may explore the effects of a similar intervention at other institutions, including whether rebalancing leads to appreciable change in training opportunities and/or workload disparities. The latter is influenced by many factors beyond panel makeup^12,16,44,45^ and will not necessarily be undone with balanced panels^12^. The need to graduate physicians prepared to care for all patients, however, is a goal of every residency program, and we suggest periodic rebalancing of panels as one tool to help achieve this goal.

## Data Availability

The data analyzed during the current study are not publicly available to protect the confidentiality of the residents involved. De-identified data may be shared upon reasonable request to the author.

## References

[1] **Fink M, Klein K, Sayers K, et al.** Objective Data Reveals Gender Preferences for Patients' Primary Care Physician. J Prim Care Community Health. 2020;11:2150132720967221. 10.1177/2150132720967221 33111633 10.1177/2150132720967221 PMC7786418

[2] **Kerssens JJ, Bensing JM, Andela MG.** Patient preference for genders of health professionals. Soc Sci Med. 1997;44(10):1531-40. 10.1016/s0277-9536(96)00272-9 9160442 10.1016/s0277-9536(96)00272-9

[3] **Schnatz PF, Murphy JL, O'Sullivan DM, Sorosky JI.** Patient choice: comparing criteria for selecting an obstetrician-gynecologist based on image, gender, and professional attributes. Am J Obstet Gynecol. 2007;197(5):548 e1-7. 10.1016/j.ajog.2007.07.025 17980206 10.1016/j.ajog.2007.07.025

[4] **Fennema K, Meyer DL, Owen N.** Sex of physician: patients' preferences and stereotypes. J Fam Pract. 1990;30(4):441-6. 2324696

[5] **Amir H, Beri A, Yechiely R, Amir Levy Y, Shimonov M, Groutz A.** Do Urology Male Patients Prefer Same-Gender Urologist? Am J Mens Health. 2018;12(5):1379-1383. 10.1177/1557988316650886 27222116 10.1177/1557988316650886 PMC6142168

[6] **Sandhu H, Adams A, Singleton L, Clark-Carter D, Kidd J.** The impact of Gender Dyads on Doctor-patient Communication: a Systematic Review. Patient Educ Couns. 2009;76(3):348-55. 10.1016/j.pec.2009.07.010 19647969 10.1016/j.pec.2009.07.010

[7] **Bertakis KD, Azari R.** Patient-centered Care: the Influence of Patient and Resident Physician Gender and Gender Concordance in Primary Care. J Womens Health (Larchmt). 2012;21(3):326-33. 10.1089/jwh.2011.2903 22150099 10.1089/jwh.2011.2903 PMC3298673

[8] **Aubrey C, Chari R, Mitchell BFP, Mumtaz Z.** Gender of Provider-Barrier to Immigrant Women's Obstetrical Care: A Narrative Review. J Obstet Gynaecol Can. 2017;39(7):567-577. 10.1016/j.jogc.2017.01.013 28625284 10.1016/j.jogc.2017.01.013

[9] **Graffy J.** Patient choice in a practice with men and women general practitioners. Br J Gen Pract. 1990;40(330):13-5. 2107832 PMC1371207

[10] **Garrison GM, Gentile N, Lai B, Angstman KB, Bonacci R.** Differential Experience with Men's and Women's Health Care Visits Between Male and Female Family Medicine Residents. Fam Med. 2016;48(7):546-50. 27472792

[11] **Franks P, Bertakis KD.** Physician gender, patient gender, and primary care. J Womens Health (Larchmt). 2003;12(1):73-80. 10.1089/154099903321154167 12639371 10.1089/154099903321154167

[12] **Rittenberg E, Liebman JB, Rexrode KM.** Primary Care Physician Gender and Electronic Health Record Workload. J Gen Intern Med. 2022;37(13):3295-3301. 10.1007/s11606-021-07298-z 34993875 10.1007/s11606-021-07298-z PMC9550938

[13] **Schmittdiel J, Grumbach K, Selby JV, Quesenberry CP, Jr**. Effect of physician and patient gender concordance on patient satisfaction and preventive care practices. J Gen Intern Med. 2000;15(11):761-9. 10.1046/j.1525-1497.2000.91156.x 11119167 10.1046/j.1525-1497.2000.91156.x PMC1495609

[14] **Kronzer VL, Leasure EL, Halvorsen AJ, Oxentenko AS, Bonnes SL.** Effect of Resident Gender and Surname Origin on Clinical Load: Observational Cohort Study in an Internal Medicine Continuity Clinic. J Gen Intern Med. 2021;36(5):1237-1243. 10.1007/s11606-020-06296-x 33078295 10.1007/s11606-020-06296-x PMC8131413

[15] **Blake RL, Jr.** Gender concordance between family practice residents and their patients in an ambulatory-care setting. Acad Med. 1990;65(11):702-3. 10.1097/00001888-199011000-00014 2102101 10.1097/00001888-199011000-00014

[16] **Liddell SS, Tomasi AG, Halvorsen AJ, Stelling BEV, Leasure EL.** Gender Disparities in Electronic Health Record Usage and Inbasket Burden for Internal Medicine Residents. J Gen Intern Med. 2024;10.1007/s11606-024-08861-0 38926324 10.1007/s11606-024-08861-0 PMC11576718

[17] Interactive Summary Health Statistics for Adults. National Center for Health Statistics. Accessed February 6, 2024, https://www.cdc.gov/nchs/nhis/ADULTS/www/index.htm

[18] **Thompson AE, Anisimowicz Y, Miedema B, Hogg W, Wodchis WP, Aubrey-Bassler K.** The influence of gender and other patient characteristics on health care-seeking behaviour: a QUALICOPC study. BMC Fam Pract. 2016;17:38. 10.1186/s12875-016-0440-0 10.1186/s12875-016-0440-0 PMC4815064 27036116

[19] **Ashman J, Santo, L, Okeyode, T. **Characteristics of Office-Based Physician Visits by Age, 2019. 184:2023. https://www.cdc.gov/nchs/data/nhsr/nhsr184.pdf

[20] **Willis J AB, Bazemore A, Jetty A, Petterson S, George J, Rosario BL, Scheufele E, Rajmane A, Dankwa-Mullan I, Rhee K.** The State of Primary Care in the United States: A Chartbook of Facts and Statistics. 2020

[21] **Couture A, Birstler J.** The Gender of the Sender: Assessing Gender Biases of Greetings in Patient Portal Messages. J Womens Health (Larchmt). 2023;32(2):171-177. 10.1089/jwh.2022.0333 36459624 10.1089/jwh.2022.0333 PMC10081704

[22] **Bryan M, Norton D, Birstler J, Chen G, Cruz L, Hanrahan L.** Resource Utilization Among Portal Users Who Send Messages: A Retrospective Cohort Study. WMJ. Mar 2020;119(1):26-32. 32348068

[23] **Adler-Milstein J, Zhao W, Willard-Grace R, Knox M, Grumbach K.** Electronic health records and burnout: Time spent on the electronic health record after hours and message volume associated with exhaustion but not with cynicism among primary care clinicians. J Am Med Inform Assoc. Apr 1 2020;27(4):531-538. 10.1093/jamia/ocz220 32016375 10.1093/jamia/ocz220 PMC7647261

[24] **Hilliard RW, Haskell J, Gardner RL.** Are specific elements of electronic health record use associated with clinician burnout more than others? J Am Med Inform Assoc. 2020;27(9):1401-1410. 10.1093/jamia/ocaa092 32719859 10.1093/jamia/ocaa092 PMC7647296

[25] **Gross R, McNeill R, Davis P, Lay-Yee R, Jatrana S, Crampton P. **The association of gender concordance and primary care physicians' perceptions of their patients. Women Health. 2008;48(2):123-44. 10.1080/03630240802313464 19042213 10.1080/03630240802313464

[26] **Bertakis KD, Franks P, Epstein RM.** Patient-centered communication in primary care: physician and patient gender and gender concordance. J Womens Health (Larchmt). 2009;18(4):539-45. 10.1089/jwh.2008.0969 19361322 10.1089/jwh.2008.0969

[27] **Prasad T, Buta E, Cleary PD.** Is Patient-Physician Gender Concordance Related to the Quality of Patient Care Experiences? J Gen Intern Med. Oct 2021;36(10):3058-3063. 10.1007/s11606-020-06411-y 33469761 10.1007/s11606-020-06411-y PMC8481522

[28] **Lau ES, Hayes SN, Volgman AS, et al. **Does Patient-Physician Gender Concordance Influence Patient Perceptions or Outcomes? J Am Coll Cardiol. 2021;77(8):1135-1138. 10.1016/j.jacc.2020.12.031 33632488 10.1016/j.jacc.2020.12.031

[29] **Noro I, Roter DL, Kurosawa S, Miura Y, Ishizaki M.** The impact of gender on medical visit communication and patient satisfaction within the Japanese primary care context. Patient Educ Couns. 2018;101(2):227-232. 10.1016/j.pec.2017.08.001 28823411 10.1016/j.pec.2017.08.001

[30] ACGME Supplemental Guide: Internal Medicine. Accessed August 23, 2024, https://www.acgme.org/globalassets/pdfs/milestones/internalmedicinesupplementalguide.pdf

[31] **van Oosterhout REM, de Boer AR, Maas A, Rutten FH, Bots ML, Peters SAE.** Sex Differences in Symptom Presentation in Acute Coronary Syndromes: A Systematic Review and Meta-analysis. J Am Heart Assoc. 2020;9(9):e014733. 10.1161/JAHA.119.014733 32363989 10.1161/JAHA.119.014733 PMC7428564

[32] **Wu J, Gale CP, Hall M, et al.** Editor's Choice - Impact of initial hospital diagnosis on mortality for acute myocardial infarction: A national cohort study. Eur Heart J Acute Cardiovasc Care. 2018;7(2):139-148. 10.1177/2048872616661693 27574333 10.1177/2048872616661693 PMC7614828

[33] **Call JB, Shafer K. **Gendered Manifestations of Depression and Help Seeking Among Men. Am J Mens Health. 2018;12(1):41-51. 10.1177/1557988315623993 26721265 10.1177/1557988315623993 PMC5734537

[34] **Smith DT, Mouzon DM, Elliott M.** Reviewing the Assumptions About Men's Mental Health: An Exploration of the Gender Binary. Am J Mens Health. 2018;12(1):78-89. 10.1177/1557988316630953 26864440 10.1177/1557988316630953 PMC5734543

[35] **Kasten Z, Lujan S, Jakeman B, et al.** Contraceptive use in patients with gender dysphoria who were assigned female at birth receiving care at a specialty gender-affirming clinic. J Am Pharm Assoc (2003). 2024;64(1):273-277. 10.1016/j.japh.2023.08.004 10.1016/j.japh.2023.08.004 37598885

[36] **Mercado F, Mercado M, Myers N, Hewit M, Haller NA. **Patient preferences in choosing a primary care physician. J Prim Care Community Health. 2012;3(2):125-31. 10.1177/2150131911421802 23803456 10.1177/2150131911421802

[37] **Bornstein BH, Marcus D, Cassidy W. **Choosing a doctor: an exploratory study of factors influencing patients' choice of a primary care doctor. J Eval Clin Pract. 2000;6(3):255-62. 10.1046/j.1365-2753.2000.00256.x 11083036 10.1046/j.1365-2753.2000.00256.x

[38] **Mavis B, Vasilenko P, Schnuth R, Marshall J, Jeffs MC.** Female patients' preferences related to interpersonal communications, clinical competence, and gender when selecting a physician. Acad Med. 2005;80(12):1159-65. 10.1097/00001888-200512000-00022 16306294 10.1097/00001888-200512000-00022

[39] **Bourke L.** Do people prefer general practitioners of the same sex? Aust Fam Physician. 2002;31(10):974-6. 12404841

[40] **Brown A.** Pew Research Center. Accessed August 23, 2024, https://www.pewresearch.org/short-reads/2022/06/07/about-5-of-young-adults-in-the-u-s-say-their-gender-is-different-from-their-sex-assigned-at-birth/

[41] **Thompson HM.** Patient Perspectives on Gender Identity Data Collection in Electronic Health Records: An Analysis of Disclosure, Privacy, and Access to Care. Transgend Health. 2016;1(1):205-215. 10.1089/trgh.2016.0007 28861535 10.1089/trgh.2016.0007 PMC5367477

[42] **Cicero EC, Reisner SL, Silva SG, Merwin EI, Humphreys JC.** Health Care Experiences of Transgender Adults: An Integrated Mixed Research Literature Review. ANS Adv Nurs Sci. 2019;42(2):123-138. 10.1097/ANS.0000000000000256 10.1097/ANS.0000000000000256 PMC6502664 30839332

[43] **Harner V, Moore M, Casillas B, Chrivoli J, Lopez Olivares A, Harrop E.** Transgender Patient Preferences when Discussing Gender in Health Care Settings. JAMA Netw Open. 2024;7(2):e2356604. 10.1001/jamanetworkopen.2023.56604 38372999 10.1001/jamanetworkopen.2023.56604 PMC10877454

[44] **Rotenstein LS, Fong AS, Jeffery MM, et al.** Gender Differences in Time Spent on Documentation and the Electronic Health Record in a Large Ambulatory Network. JAMA Netw Open. 2022;5(3):e223935. 10.1001/jamanetworkopen.2022.3935 35323954 10.1001/jamanetworkopen.2022.3935 PMC8948526

[45] **Rotenstein L, Jay Holmgren A.** COVID exacerbated the gender disparity in physician electronic health record inbox burden. J Am Med Inform Assoc. 25 2023;30(10):1720-1724. 10.1093/jamia/ocad141 37436709 10.1093/jamia/ocad141 PMC10531114

